# Aerosolization and recovery of viable murine norovirus in an experimental setup

**DOI:** 10.1038/s41598-020-72932-5

**Published:** 2020-09-29

**Authors:** Malin Alsved, Anders Widell, Henrik Dahlin, Sara Karlson, Patrik Medstrand, Jakob Löndahl

**Affiliations:** 1 Ergonomics and Aerosol Technology, Design Sciences, Lund University, Lund, Sweden; 2 Clinical Virology, Department of Translational Medicine, Lund University, Lund, Sweden

**Keywords:** Applied microbiology, Infectious diseases

## Abstract

Noroviruses are the major cause for viral acute gastroenteritis in the world. Despite the existing infection prevention strategies in hospitals, the disease continues to spread and causes extensive and numerous outbreaks. Hence, there is a need to investigate the possibility of airborne transmission of norovirus. In this study, we developed an experimental setup for studies on the infectivity of aerosolized murine norovirus (MNV), a model for the human norovirus. Two aerosol generation principles were evaluated: bubble bursting, a common natural aerosolization mechanism, and nebulization, a common aerosolization technique in laboratory studies. The aerosolization setup was characterized by physical and viral dilution factors, generated aerosol particle size distributions, and the viral infectivity after aerosolization. We found a lower physical dilution factor when using the nebulization generator than with the bubble bursting generator. The viral dilution factor of the system was higher than the physical dilution; however, when comparing the physical and viral dilution factors, bubble bursting generation was more efficient. The infectivity per virus was similar using either generation principle, suggesting that the generation itself had a minor impact on MNV infectivity and that instead, the effect of drying in air could be a major reason for infectivity losses.

## Introduction

The hypothesis that the human norovirus (NoV) to some extent can be spread through air is gaining more support as recent studies detected NoV RNA in air during outbreaks in hospitals^1^, close to symptomatic patients^2^, and after simulated floor cleaning procedures^3^. Possible sources of airborne NoV in real-life settings are bubble bursting during vomiting and toilet flushing^2,4^ or resuspension from contaminated surfaces^3^. Our recent findings showing that aerosol particles smaller than 1 µm in diameter contain detectable amounts of NoV RNA indicate that viruses can travel longer distances than splashing droplets (2 m) and also remain airborne for longer periods of time^2^. Today, there are only scarce estimates of the importance of airborne NoV contribution to the overall transmission of the disease^5^. A key knowledge gap concerns the ability of viruses to preserve their infectivity in air under different conditions. Improved knowledge of this issue is necessary to establish effective measures for disease control.

In order to address this issue, information on parameters that influence infectivity of airborne noroviruses is crucial. However, the infectivity of airborne human noroviruses has not been possible to assess in real-world environments, nor in laboratory experiments, since there is no well-established cell culturing system for these viruses working at the low concentrations obtained in air samples^6^. To be able to study NoV viability, the cultivable murine norovirus (MNV) is frequently used as a model^7,8^. Moreover, MNV infections are a common problem in mice in laboratory animal facilities^9^.

Evaluations of viral infectivity after aerosolization have been performed in previous studies^1,10–17^. Yet, many of these studies lack a detailed representation of the generated aerosol, such as size characterization from nanometer to micrometer sized aerosol particles, physical dilution factor (or spray factor) of the setup, and the effect of drying on particle size^18^. The aerosol particle size is important since it influences the residence time in air, the amount of protective material surrounding the viruses, and in some cases also the severity of the infection in the host^19^. Several studies report detection of viruses in airborne sub-micrometer particles^2,20–22^. These smaller aerosol particles may be of special importance in disease transmission as they may remain airborne for long periods of time (hours) and easily deposit in the respiratory tract during inhalation. Thus, there is a need for more research targeting such small aerosol particles.

To our knowledge, only one previous study has evaluated the infectivity of aerosolized MNV^1^. In that study, a twin fluid nebulization technique was used, which is the most common type of bioaerosol generators in laboratories. A range of such devices is available with different specifications, the Collison nebulizer being the most common^18^. Twin fluid nebulization is an efficient aerosolization technique, but its resemblance to natural aerosol generation processes is limited. On the other hand, twin fluid nebulization devices are easy to operate, have a high constant aerosol output, and consume little material due to recirculation. A frequent dilemma is that gentle aerosolization mechanisms result in lower particle concentrations, which in turn leads to low sample concentrations. To counter this problem, a device that simulates the natural mechanism of bubble bursting was developed^23,24^; the sparging liquid aerosol generator (SLAG). The SLAG has been shown to preserve bacterial membrane integrity better than nebulization^25,26^, while still generating a high aerosol particle concentration^27–30^. Although most enteric viruses are not enveloped, a recent study suggested that rotaviruses and noroviruses can transport within vesicles^31^, thus there may be reason to choose an aerosolization technique that preserves such structures.

This study aim to provide a well-characterized experimental setup for aerosolized MNV. Two aerosol generators were compared: the SLAG employing bubble bursting, and the atomizer employing twin fluid nebulization. The aerosols were characterized by the complete particle size distribution from 10 nm to 10 µm. The physical and viral dilution factors were determined using a radioactive tracer molecule and viral genomes, respectively. For infectivity assessment of the aerosolized MNV, negative sense RNA (nsRNA) detection in cell cultures was optimized for the purpose and used for the collected samples.

## Results

**Figure 1:**
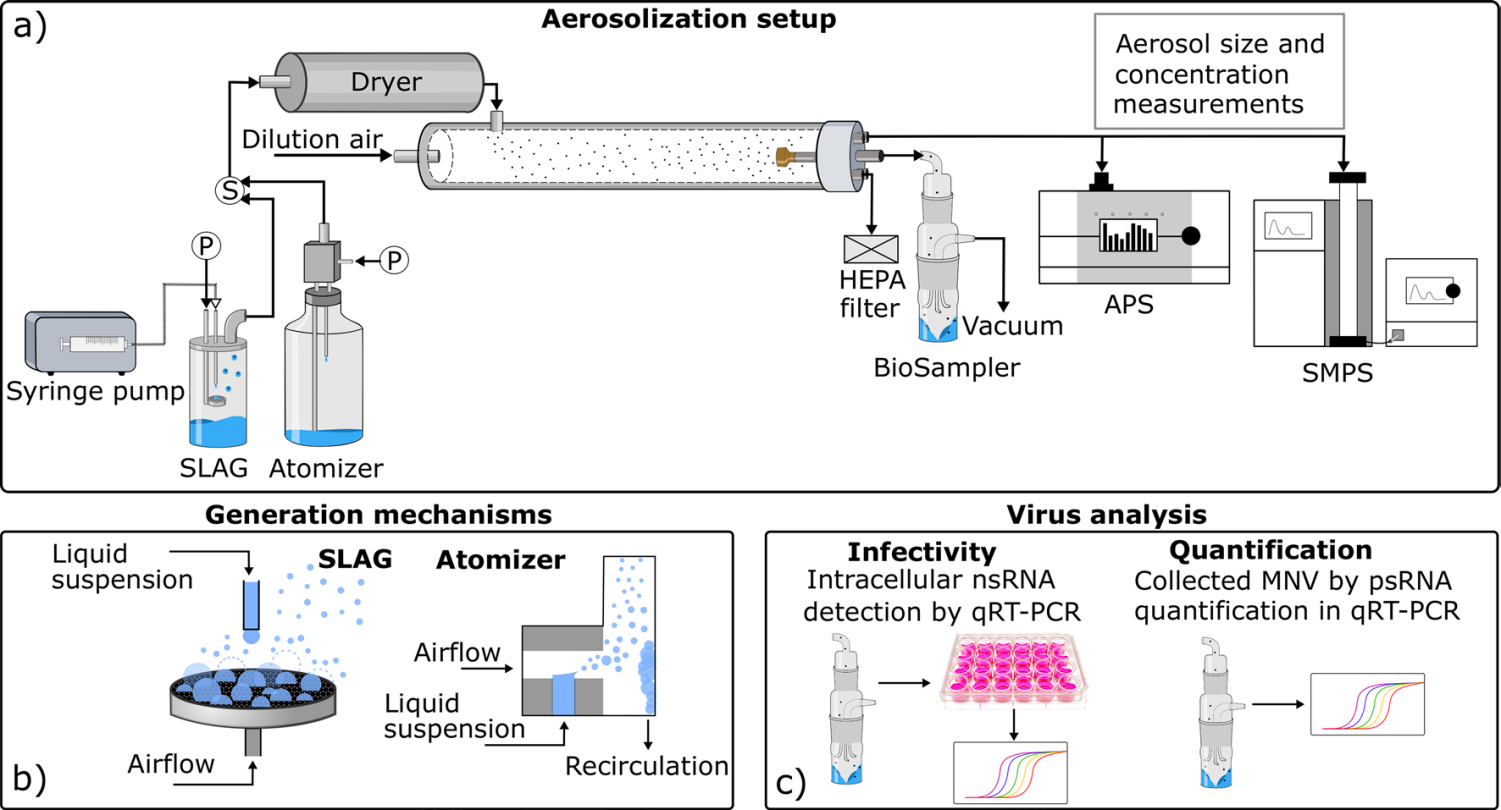
Experimental setup for aerosolization and analysis of murine norovirus. The aerosolization setup used for experiments on airborne murine noroviruses (**a**). Black arrows represent the aerosol flow, and the circled ‘P’s indicates where pressurized air was connected to the aerosol generators. The aerosol was dried in a diffusion dryer before entering the flow tube, where it was diluted with particle-free air. At the end of the flow tube, the aerosol particle concentration was monitored with a scanning mobility particle sizer (SMPS) and an aerodynamic particle sizer (APS), and collected into a BioSampler impinger for analysis. All ports for excess air at the end of the flow tube were connected to HEPA filters. The aerosol generation mechanisms for the SLAG and the atomizer are outlined in **b**). The collection liquid in the BioSampler was analyzed by a viral infectivity assay and by viral RNA quantification, outlined in **c**). SLAG: Sparging liquid aerosol generator; HEPA filter: high efficiency particulate air filter; nsRNA: negative sense RNA; psRNA: positive sense RNA; MNV: murine norovirus; qRT-PCR: quantitative reverse transcriptase polymerase chain reaction.

We developed an experimental setup for aerosolization and collection of MNV with subsequent infectivity assessment in cell cultures (Fig. 1). MNV was aerosolized through either bubble bursting (by the SLAG) or twin fluid nebulization (by the atomizer). The generated liquid aerosol droplets were dried to droplet nuclei particles in the air, similar to aerosol droplets formed in real-world settings in air at regular relative humidity (RH < 70%). The MNV containing aerosol was collected in a liquid impinger and analyzed for infectivity by inoculation of permissive cell cultures. Detection of intracellular nsRNA by strand specific quantitative reverse transcription (qRT) PCR in these cell cultures was used as a measure of virus infectivity since the nsRNA is specific for replication. The viral dilution factor was determined by qRT-PCR targeting the MNV genome – the positive sense RNA (psRNA) – in the starting liquid and the collection liquid. The physical dilution factor of the aerosolization setup was evaluated using a radioactive tracer (^99m^Technetium).

### Aerosol particle size characterization

**Figure 2:**
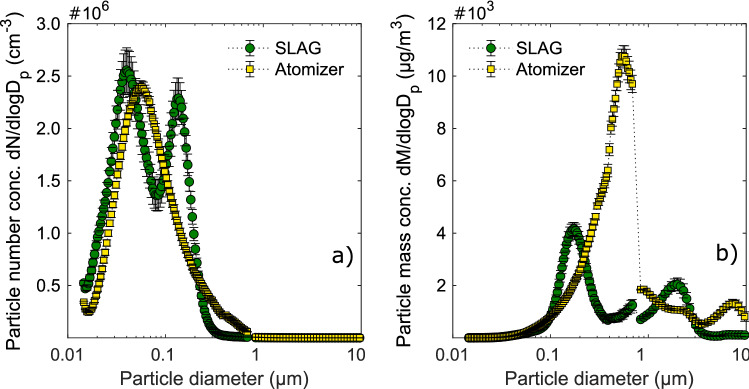
Aerosol particle size distributions generated by bubble bursting and nebulization. Aerosol particle size number distribution (**a**), and mass distribution (**b**), generated from the SLAG and the atomizer. The aerosolization solution contained 90% PBS and 10% MNV stock solution (MNV in supplemented growth media). The count median diameter (CMD) for the SLAG aerosol was 0.080 µm and for the atomizer aerosol 0.092 µm. The mass median diameter (MMD) for the SLAG aerosol was 0.89 µm and for the atomizer aerosol 1.1 µm. Each data set represents an average of continuous measurements during three replicate experiments. SLAG: sparging liquid aerosol generator.

The aerosol particle size number distribution (Fig. 2a) shows that by number, the majority of the dry aerosol particles were < 0.2 µm in diameter using either the SLAG or the atomizer, with a count median diameter of 0.080 µm and 0.092 µm, respectively. When instead comparing the aerosol particle size mass distributions (Fig. 2b), the majority of the particle mass is found in particles > 0.1 µm. The mass median diameter (MMD) of the aerosol generated by the SLAG and atomizer was 0.89 µm and 1.1 µm, respectively. The bimodal size distribution from the SLAG was expected, as there are two mechanisms for droplet formation in bubble bursting: film drops (first size mode) and jet drops (second size mode)^32^.

Ammonium sulphate particles aerosolized from a solution with the same setup allowed for the analysis of the original droplet size from the generators. Using the dry aerosol particle sizes which we could measure, calculations were made to extrapolate the sizes of the wet droplets initially generated (Eqs. 1 and 2 in the Methods section). The ammonium sulphate solution contained 1% salt. Hence, the volume decreased by ~ 200 times when the water evaporated from the droplet to form dry droplet nuclei particles. As a result, the diameters of the dry particles were about six times smaller than the initial wet droplet particles, changing the particle size range from 0.06 to 30 µm (wet) to 0.01–5 µm (dry). The phosphate buffered saline (PBS) contained 0.9% salt; thus, the change of size during drying is expected to be comparable with the MNV aerosolization.

### Physical and viral dilution factors of the system

The radioactive nuclei ^99m^Tc was used as a tracer molecule in the aerosolized solution to allow for high-resolution determination of the dilution factor from starting liquid to collection liquid in the setup. The collection of aerosol generated by the SLAG resulted in a physical dilution factor of 2.4 log_10_, three times higher than the dilution factor for the atomizer, which was 1.9 log_10_ (mean of triplicate runs).

Aerosolization of ^99m^Tc was also used to evaluate the collection efficiency of the BioSampler for the generated aerosols by comparison with collection on filters. Relative to the particle filters, the BioSampler collected 66% and 84% of the particle volume generated by the SLAG and the atomizer, respectively. The lower collection efficiency of the SLAG aerosol can be explained by the larger amount of particle mass below 0.5 µm (Fig. 2b) that is not efficiently collected by the BioSampler^11^. This difference in particle size can also explain the higher physical dilution factor observed for the SLAG aerosol than for the atomizer aerosol.

The viral dilution factor was determined as the psMNV concentrations in the start solution compared to the collection solution. For both aerosol generators, the viral dilution factors were higher than the physical. However, the difference was threefold for the SLAG aerosol while eightfold for the atomizer aerosol. Taken together, as compared to the atomizer, a smaller amount of SLAG aerosol is collected by the BioSampler (i.e., higher physical dilution), but these particles contain more MNV psRNA copies (lower viral dilution relative the physical dilution).

### Negative sense RNA detection in infected RAW 264.7 cells

Detection of nsRNA by qRT-PCR in cell homogenates was performed with a limit of detection of 50 RNA copies per reaction (Cycle threshold; Ct ~ 40). Both the positive and negative strand specific qRT-PCR assays were run with their respective opposite sense RNA standards to determine the concentrations needed to avoid false priming amplification. Using the negative sense primers, concentrations of psRNA above 5 × 10^6^ copies per reaction resulted in Ct values ~ 40. The corresponding Ct-value for falsely detected nsRNA using the positive sense primers was 33 for an nsRNA concentration of 5 × 10^6^ copies. As noted by Vashist et al.^33^, unspecific priming could occur from the RT enzyme itself, which showed up in our analysis as a secondary melting temperature in the melting curve in samples with low template RNA. Thus, samples without the correct melting temperature were discarded and a cut-off at Ct 40 was used.

### Optimization of incubation time for negative sense RNA detection

**Figure 3:**
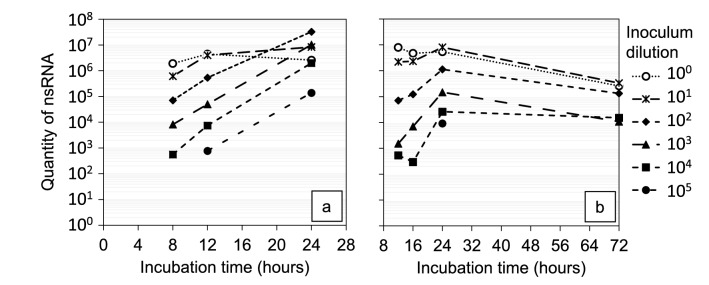
Negative sense RNA concentrations in cells infected with murine norovirus. Intracellular nsRNA concentrations in wells inoculated with serially diluted MNV stock solution after a variety of incubation times. Two separate experiments were conducted: one with the incubation times 8, 12 and 24 h, and one with the incubation times 12, 16, 24 and 72 h. Results are based on duplicate infected wells at each level.

Optimal incubation time for nsRNA detection inside the infected cells was evaluated by a time series analysis of the nsRNA concentrations. The nsRNA concentration increased with longer incubation time up to 24 h (Fig. 3a). A maximum nsRNA was reached at 12 h for the wells inoculated with the highest viral load due to rapid cell death (verified by microscopy). The cells inoculated with the lowest viral concentration (10^5^ dilution factor) displayed nsRNA quantities close to or below the limit of detection after 8 and 12 h incubation. Therefore, the incubation times 16 h and 72 h were evaluated in a second time series experiment (Fig. 3b). However, 16 h also resulted in undetectable nsRNA values with the 10^5^ diluted inocula and 72 h resulted in decreased nsRNA concentrations for all dilutions compared to 24 h. Thus, 24 h was chosen as the optimal incubation time for the low infectivity aerosol samples.

**Figure 4:**
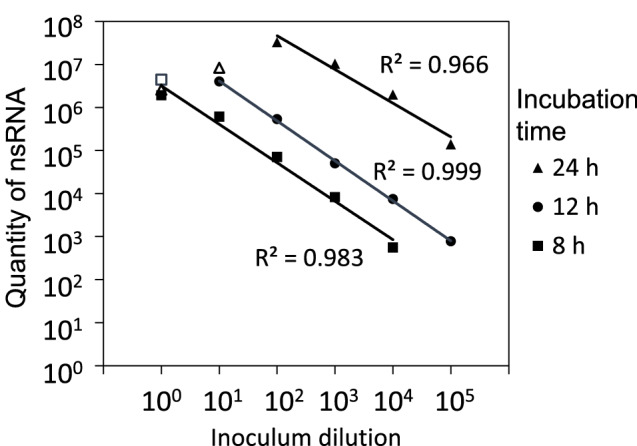
Linear regression analysis of negative sense RNA in cells depending on inoculum dilution. nsRNA concentration in wells inoculated with serially diluted MNV stock solution, after the incubation times 8, 12 and 24 h. Linear regression lines (solid lines) were fitted to the log-transformed data. Open data points (12h_10^0^, 24h_10^0^, 24_10^1^) were not included in the fit. The p-values were *p* < 0.01 for the 8 and 12 h regression lines, and *p* < 0.05 for the 24 h regression line.

Regression lines were fitted to the same data as in Fig. 3a, for evaluation of the relation between nsRNA quantities and the viral inoculation concentration after the different incubation times (Fig. 4). Here, the samples that had already reached maximum nsRNA quantities (10^0^ after 12 h, and 10^0^ and 10^1^ after 24 h) due to cell death were excluded from the fit. Significant correlations between nsRNA quantity and viral inoculation concentration were found for all three incubation times (*p* < 0.01 for 8 and 12 h, and *p* < 0.05 for 24 h). The comparison of the three time points shows a 1 log nsRNA increase every 4 h during the infection phase.

### Infectivity of aerosolized murine norovirus

**Figure 5:**
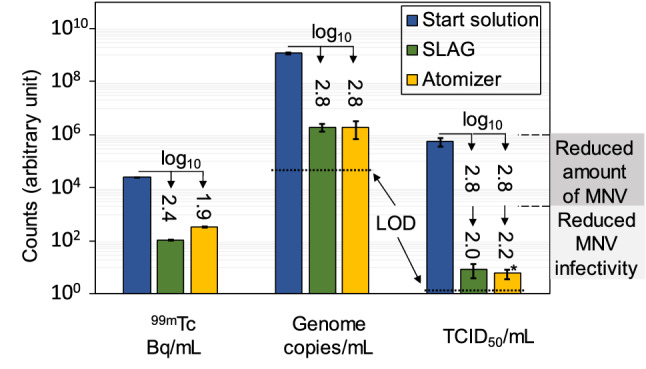
Comparisons of the three measured entities in the start solution and the collection liquids. Comparison of concentrations and MNV infectivity in the start solution before aerosolization with that of the material collected in the BioSampler. Each set of three bars has its unit below, and the y-axis therefore only provides the magnitude. Arrows are included with the corresponding logarithmic dilution factors. Dotted lines indicate the limit of detection (LOD) for the genome detection and TCID_50_ assay. The LOD for the ^99m^Tc analysis was below the scale of this chart. The mean concentrations of ^99m^Tc radioactivity and MNV genomes are based on triplicate collected aerosol samples. The TCID_50_ mL^−1^ concentrations are the means of three aerosol collections and triplicate infected wells for each sample. The start solution mean is based on three infection plates with triplicate infected wells. *One of three replicates did not reach the TCID_50_.

Virus infectivity after aerosolization and collection were evaluated by cell inoculations with serially diluted aerosol samples. Each inoculated cell culture was assessed qualitatively for infectivity by detection of intracellular nsRNA, and was then used to calculate the 50% tissue culture infectious dose (TCID_50_) mL^−1^ (Fig. 5). The TCID_50_ mL^−1^ values were similar for the MNV from both generators, which agrees well because the genome concentrations (psRNA) were also analogous. The 2.8 log_10_ viral dilution factor, together with the viral infectivity loss, resulted in a 5 log_10_ factor reduction in TCID_50_ mL^−1^. The viral infectivity loss per viral genome copy was, hence, reduced a factor of ~ 2 log_10_ for both aerosol generators.

## Discussion

Understanding the mechanisms by which viral diseases can be transmitted through air is of importance for the development of accurate infection prevention guidelines and practices. Noroviruses, which traditionally have not been considered to transmit via air, have nevertheless been detected in air samples^1,2,34^. This gives reason to study the ability of noroviruses to maintain infectivity after aerosolization. In this study, we developed a methodology for studying infectivity of aerosolized MNV, a model virus for human norovirus. We observed that viable virus could be transferred but that the infectivity levels were reduced by two orders of magnitude after airborne transport.

The aerosolization setup developed in this study has similarities with previously described systems^1,11,12,25,35–38^, however our characterization of the setup is extensive and contributes detailed information on system performance. We also evaluated two aerosol generators for usage in aerosolization studies on viruses, since their aerosolization mechanisms are physically different: the SLAG simulates natural bubble bursting, while the Collison type atomizer utilizes twin-fluid nebulization frequently used in laboratory studies. In addition, we used a radioactive tracer to obtain the physical dilution factor with higher precision than with common chemical tracers, and an alternative method for validation of infected cell cultures using negative strand specific qRT-PCR.

Previous studies have focused either on the physical dilution factor^39,40^ or the viral genome dilution factor (together with infectivity reduction)^12,14,15^. Here we compared these two, and found that the physical dilution factors for both aerosol generators were lower than the viral dilution factors (Fig. 5). Both MNV and radioactive tracer were mixed in the same starting solution and thus, the observed disparity could be due to an uneven distribution of MNV in the droplets. As the atomizer is based on shear forces, supposedly both the radioactive molecule and the virus should be evenly distributed in the liquid, and hence, also in the generated aerosol droplets, as has been shown by Pan et al.^15^. Another reason could be due to losses of MNV that stick to the BioSampler glass container surfaces, while ^99m^Tc molecules to a higher degree remain dissolved in the sampling liquid.

Compared to the atomizer, the SLAG aerosol was collected less efficiently by the BioSampler due to the generation of fewer large particles (Fig. 2a). This was also demonstrated by a larger physical dilution of the radioactive tracer (Fig. 5). Nevertheless, the viral dilution factors for both generators were similar. Thus, the bubble bursting aerosolization appears to be more efficient than the atomizer in generating virus-containing particles that are collected. Presumably, a higher concentration of MNV in larger droplets could result in this. The physics and fluid mechanics behind bubble bursting in non-clean water have recently been investigated by Poulain and Bourouiba^41^, showing that surfactants released from microorganisms may enhance aerosol droplet formation upon bubble bursting. If the same mechanisms can explain enhanced virus aerosolization from bubble bursting remains to be explored.

The SLAG has only been used for aerosolization of influenza virus in one previous study^36^, but then no infectious virus was detected in the collected samples. This may be due to a lower emission rate of the SLAG compared to most atomizers. In our study, the atomizer generated approximately the same amount of aerosol at an airflow rate of 2 L min^−1^ as the SLAG at 10 L min^−1^.

The MNV (about 30 nm in diameter) is at least one order of magnitude smaller than the generated aerosol droplets and do seemingly not influence the droplet size distribution^11,15^. The majority of the droplets generated here were smaller than 1 µm. We have shown in a previous study that human norovirus can be present in sub-micrometer particles in hospital wards during outbreaks^2^. Hence, there is a need to also include also these smaller particles in studies on viral aerosolization.

Considering that the majority of bioaerosol particles in the environment are 1–10 µm in diameter^42^, most aerosol samplers have their highest collection efficiencies in that size range. However, viruses have been found in sub-micrometer particles both in the environment and in laboratory studies^2,20,22,36,43,44^, thus, a higher collection efficiency for small particles would greatly improve studies on aerosolized viruses. A recently developed technology for bioaerosol collection based on condensational growth has been shown to have > 95% collection efficiency for sub-micrometer aerosol particles, which would give a more representable result as viruses in all particle sizes are included in the downstream analysis^15^. The condensational growth collector has a liquid collection volume that is ten times smaller than that of the BioSampler, which would increase the virus concentration in the collected sample. This up-concentration would be advantageous for further studies on virus infectivity using the setup, because the infectivity detection assay presented here was close to the detection limit.

The decreased MNV infectivity after aerosolization in this setup was reduced almost to the same amount (comparing infectivity/genome copy number) by the SLAG and the atomizer, which suggests that for this MNV, the aerosolization mechanism had little impact on infectivity. The atomizer recirculated the liquid, which is known to degrade biological cell membranes^25^ and cause water to evaporate^37^. Previous studies also did not find any loss of viability in MNV after 25 min recirculation in a Single-Jet atomizer^1^, and in coronavirus after recirculation in a Collison nebulizer^37^, even though coronaviruses are enveloped viruses. Instead, it has been shown that drying can be detrimental for non-enveloped viruses^10^. Moreover, viruses aerosolized from liquids with low-solute concentrations showed reduced infectivity^10^. These two parameters, drying and low-solute solution, could explain the difference in MNV infectivity observed here and in the previous study by Bonifait et al.^1^. Bonifait et al. used an aerosol generator that produced droplets around 1.5 µm in diameter, which is about 7 times larger in diameter (> 100 times larger volume) than the droplets produced in this study. They also aerosolized MNV in virus growth medium, while in our study, MNV in growth media was diluted 1:10 in PBS (due to foaming in the SLAG).

Drying of MNV in laboratory settings has also been performed on surfaces^45,46^. These studies found that MNV dried in larger droplets (100–200 µL) or at higher relative humidity was more likely to be inactivated, and it was hypothesized that the drying time, rather than the fact that water was removed, was the likely reason for MNV inactivation^45^. These results taken together, suggest that MNV is likely to remain infectious if dried fast (however, not completely) in a solution with protective molecules (proteins and sugars).

Samples collected from aerosols often exhibit low viral concentrations^12^ and therefore require a method with high sensitivity for infectivity detection. A previous study developed a method for molecular detection of an infection-specific molecule for MNV: the negative strand RNA (nsRNA), which is the complementary strand to the viral genome^33^. The nsRNA is produced inside cells exclusively during ongoing replication, thereby serving as a qualitative marker for infection. This method can result in fewer false negatives than the conventional observation of the cytopathic effect (CPE) in cell cultures, since the sensitivity of qRT-PCR is high. Other advantages are that the molecular analysis is objective, and that it does not require personnel with long experience of cell work. However, using nsRNA detection as a qualitative determiner still requires inoculation with serial dilutions to obtain a quantitative value of infectivity, such as the TCID_50_.

The risk for false priming of the opposite sense RNA in the qPCR step was investigated by adding psRNA to the nsRNA assay (primers for nsRNA), and vice versa. Since we needed to add a high psRNA concentration of 5 × 10^6^ copies per reaction to reach a borderline positivity (Ct value of 40), the specificity of the key nsRNA assay is sufficiently high. In addition, Vashist et al. showed that including both negative and positive sense RNA in the RT step for cDNA synthesis did not alter the qPCR results for either set of primers. Unspecific priming of the RT enzyme occurred only when there was no amount or too low amounts of RNA template and could be identified by lower melt temperatures; hence, samples with aberrant melting temperatures were not included in the analysis.

Santiana et al. showed that MNV replication occur mainly within 24 h of incubation, while 90% of the cells were still intact^31^. Most of the cell membrane damages took place after 24 h and during this period MNV replication was limited. This pattern is in agreement with our results that show the highest nsRNA concentrations around 24 h, and then decreasing amounts due to cell death (Fig. 3). In addition, the time series analysis can be used for estimates of the infectivity of samples by simply harvesting cells at 24 h, purify and amplify nsRNA and plot the nsRNA Ct values against a standard curve. This would simplify comparisons in future studies of incremental changes in aerosol parameters (humidity, temperature, etc.). To observe a CPE in cell cultures, an incubation time of 72 h was needed. However, after 72 h, many cells were destroyed and the intracellular nsRNA was degraded. As the infectivity was strongly reduced after aerosolization, the nsRNA detection after 24 h was chosen instead of CPE observation after 72 h.

The experimental setup described here highlights some difficulties in studies on aerosolized viruses: (1) the lack of standards in how to generate bioaerosol that results in significant differences in aerosol particle size and concentration, (2) the necessity to determine both physical and viral dilution factors, and (3) the low viral infectivity of the collected virus after aerosolization which may be explained by a combination of extensive drying during airborne transport due to the low-solute solution and dilution in the setup. By thorough characterization and description of the system performance and the aerosol characteristics, we provide new information on sub-micrometer aerosol particle generation and the effects on MNV infectivity. In addition, our results suggest that virus aerosolization by bubble bursting may be advantageous. The aerosolization setup developed in this study allows for further analysis of how the infectivity of airborne MNV is affected by factors in the environment, such as temperature, relative humidity and aerosol particle size. This may lead to a deeper understanding of seasonal and regional differences in virus transmission and offer better tools to minimize the spread of viral aerosols, and thus, prevent spreading of diseases.

## Methods

### Experimental aerosolization setup

An experimental setup for aerosolization and collection of viruses was developed (Fig. 1), where either a Collison type atomizer (Constant Output Atomizer, model 3076, TSI Inc.) or a SLAG (Sparging Liquid Aerosol Generator, CH Technologies) was used as the aerosol generator. The atomizer generated droplets by using a high perpendicular airflow to break up a thin liquid column (see schematic in Fig. 1, lower left panel). The largest droplets impacted on the wall inside the generator and were recirculated. In the SLAG, the solution to be aerosolized was dropped onto a porous stainless steel plate at a flow rate of 0.5 mL min^−1^ delivered by a syringe pump. At the same time, air was blown through the porous plate from below, bubbling through the liquid film of the MNV suspension, generating droplets though bubble bursting (see schematic in Fig. 1, lower left panel). The atomizer was operated at 1.2 bar and a 2 L min^−1^ output airflow rate, and the SLAG at a 10 L min^−1^ output flow rate. The generated aerosols were dried in a silica diffusion dryer (DDU 570, Topas GmbH) and then mixed with a perpendicular dilution flow in a stainless steel flow tube to a total airflow rate of 18 L min^−1^. At this flow rate the particles were airborne about 10 s before collection at the end of the flow tube. Aerosol particles were collected by a BioSampler impinger (SKC Inc.), with 20 mL PBS as collection liquid, at an airflow rate of 12.5 L min^−1^.

### Aerosol particle size characterization

The particle number concentration in the air was monitored by an Aerodynamic Particle Sizer (APS, model 3321, TSI Inc.) and a Scanning Mobility Particle Sizer (SMPS, consisting of a Differential Mobility Sizer, model 3080, TSI Inc. and a Condensational Particle Counter, model 3775, TSI Inc.). The APS measured particles in the size range 0.8–20 µm and the SMPS in the size range 0.014–0.68 µm.

Characterization of the initial droplet sizes of the generated aerosol was performed using a 1% ammonium sulphate solution, which was chosen because it forms spherical particles when dried^47^. Using the measured dry salt aerosol particles *d*_*dry*_, the originally generated droplet diameters *d*_*wet*_ were calculated with Eqs. 1 and 2:

1 $$m_{wet} \cdot f_{salt} = m_{dry}$$

2 $$d_{wet} = d_{dry} \cdot \sqrt[3]{{\frac{{\rho_{dry} }}{{\rho_{wet} \cdot f_{salt} }}}}$$

where *m*_*wet*_ is the mass of the wet droplet, *m*_*dry*_ is the mass of the dry particle, *f*_*salt*_ is the mass fraction of salt in the solution, and *ρ*_*dry*_ and *ρ*_*wet*_ are the densities for dry particles and wet droplets. The wet droplet diameters range was 0.060–80 µm, with a gap between 3.4 and 5.0 µm where the instrumental measurements did not overlap.

### Physical dilution factor by radioactive tracer

The physical dilution factor of the aerosolization setup was determined by aerosolization of radioactive ^99m^Tc in a solution of PBS and 10% growth media containing MNV (same solution properties as those used for MNV aerosolization). Three runs of 30 min aerosolization were performed with each aerosol generator and triplicate (1 mL) aliquots were used for the analysis. The same start solution was used for both generators.

Gamma-ray spectroscopy was used to measure the radioactivity from ^99m^Tc by means of a sodium iodine well count detector (1480 Wizard, Perkin Elmer). The gamma radiation emitted from the start solution was then compared to that of the collection liquid. In addition, PBS was analyzed as a reference. The measuring time for each sample was set to 90 s, and the results were adjusted for radioactive decay during the measurement time. The instrument automatically corrected for background activity. All samples were below the max count rate of 2 million counts per minute, where the dead time error is < 1%.

The collection efficiencies of the BioSampler for the two generated aerosols were evaluated by parallel collection of radioactive aerosol particles on a 25 mm HA filter with 0.45 µm pore size (Millipore). The filter sampled close to 100% of all particles at a flow rate of 1 L min^−1^ (data not shown). Filters were placed in sample tubes (same as for liquid samples) with a pincer and analyzed together with the liquid samples by gamma-ray spectroscopy. For comparison, the radioactivity from the BioSampler and the filter were normalized by their respective sampling airflow.

### Aerosolization of murine noroviruses

The MNV stock solution in growth media was diluted 1:10 in PBS to decrease foaming due to high viscosity (primarily problematic in the SLAG) before aerosolization. The start solution was inserted into the atomizer flask or the syringe connected to the SLAG, and aerosolization with collection into the BioSampler was run for 30 min. Aerosolization was performed in triplicates for each aerosol generator. Samples were taken from the start solution before the experiment and from the collection liquid in the BioSampler after the experiment. Samples were stored in − 80 °C freezer until analysis was performed, either by the MNV infectivity assay or by qRT-PCR for quantification (viral dilution factor).

### Murine norovirus preparation

The MNV strain Berlin/06/06DE S99 was cultured in the permissive murine cell line RAW 264.7 grown in Dulbecco’s minimum essential medium (DMEM, no pyruvate; catalogue no. FG 0435, Biochrom), supplemented with 10% low endotoxin foetal bovine serum (Hyclone FBS, Nordic Biolabs), 1% non-essential amino acids (Life Technologies) and 5% penicillin/streptomycin (Life Technologies). Cell growth flasks were purchased from Corning Life Sciences and 24-well cultivation plates (TPP Techno Plastic Products AG) from Sigma Aldrich.

Cells were grown in 6 mL media at 37 °C and 5% CO_2_. The cell culture was split three times per week by gently scraping cells off the flask bottom, and transferring 1/5 of the cells to fresh growth media in a new flask. The cell concentration before every split was determined by Trypan blue staining (1:1) and cell counting (EVE Automated Cell Counter, Nanoentek).

### Infectivity analysis of MNV

Prior to viral inoculations in 24-well plates, 800,000 freshly split cells were added per well (in 400 µL media) and allowed to grow overnight to a monolayer. After the incubation, the supernatant from each well was removed and the cells were washed with PBS. Viral tenfold dilution series in PBS were prepared and 400 µL were added to each well and incubated for 1 h, and were then replaced by fresh growth medium and incubated for the assigned incubation time (24 h for aerosol samples). After incubation, the supernatant was removed and the cells were washed with PBS, and thereafter lysed with 350 µL RLT lysis buffer (RNeasy Mini Kit, Qiagen, Inc.) and all samples were stored at − 20 °C.

Intracellular RNA was extracted from the samples using the RNeasy Mini Kit (Qiagen, Inc.) and then treated with DNase (Thermo Fisher Scientific) for 30 min at 37 °C, according to the protocol of the manufacturer. Extracted RNA samples were stored at − 80 °C.

### Strand specific quantitative reverse transcription PCR

During replication of the genomic psRNA, MNV produces an intermediate stage of nsRNA that is used for generating multiple copies of psRNA. The number of nsRNA copies is usually orders of magnitudes lower than the number of psRNA copies. Hence, detection of the nsRNA, unique for viral replication, calls for a specific reverse transcription step starting from a primer that is uniquely hybridizing to the negative strand. For MNV, such a strand-specific method has been described by Vashist et al.^33^ and shown to be specific. We have used this methodology here (as well as in previous studies^46^). In short, the transcription primer targeting the nsRNA has an added non-viral oligonucleotide sequence in its 5′-end. In the subsequent qPCR reaction, a primer identical to the non-viral oligonucleotide tag is used in order to only amplify what was transcribed using the tagged primers. A similar reverse tagged transcription step was used to target the more abundant psRNA. Both negative and positive strand transcriptions were conducted with SuperScript IV in a thermal cycler (Applied Biosystems 2720) according to previously developed methodology^33,46^. These samples containing cDNA transcripts were stored at − 20 °C until quantification by qPCR. SYBR Select Master Mix (Thermo Fisher Scientific) was used for qPCR in 20 µL reactions using a StepOnePlus Real-Time PCR system (Applied Biosystems). For relative quantification, serially diluted positive sense and negative sense cDNA respectively, with a concentration of 2.5 × 10^6^ copies µL^−1^ were included in the qPCR setup. The data was analyzed in StepOne Software v 2.3.

### Determining the TCID_*50*_ of the aerosolized samples

Detection of nsRNA by qRT-PCR was used as a qualitative measure of infection in each well sample and used to calculate the TCID_50_ mL^−1^ ^48^. The TCID_50_ was determined per aerosolization run (three per aerosol generator) based on triplicate wells per dilution, and the mean and standard deviation of these were used for comparisons. The TCID_50_ mL^−1^ was correspondingly determined for the start solution.

### Time series analysis of intracellular negative sense RNA

To find the optimal incubation time for viral samples with low concentrations (i.e., the collected aerosol samples) a time series analysis was performed to evaluate the nsRNA concentration at various incubation times. The MNV stock solution was diluted in growth media in tenfold dilution series, ranging from zero to 10^10^ times dilution. Three 24-well plates were inoculated with viral solution (duplicate wells) as described above. After the inoculation, the plates were incubated with fresh media for 8, 12 and 24 h, respectively. After the assigned incubation time, the supernatant was removed and the cells were lysed as above. The plates were stored at − 20 °C until RNA extraction and further analysis by qRT-PCR for detection and quantification of nsRNA, as described above. A second time series analysis experiment was performed with the incubation times 12, 16, 24 and 72 h.

### Statistics

Aerosolization and collection were performed in triplicate runs for each aerosol generator, and the mean and standard deviation for each set of triplicate runs were calculated. The dilution factors were calculated by dividing the mean value of the start liquid concentration by the mean value of the collection liquid concentration. Triplicate aliquots were extracted from the collection liquids for the ^99m^Tc analysis, as well as for the psRNA quantification, and the means of those were used to represent one sample. The qPCR analysis was performed using duplicate reactions and the mean of those was used. Linear regression analysis using the ANOVA test on the log-transformed data was used to compare the quantities of nsRNA with inoculation dilutions in Fig. 4.
